# Discrimination of atrial fibrillation burden using cardiac magnetic resonance imaging

**DOI:** 10.1016/j.hroo.2026.03.011

**Published:** 2026-03-19

**Authors:** Andreas U. Gasser, Stefanie Aeschbacher, Michael Coslovsky, Vincent Meier, Tanja Ruoff, Tobias Reichlin, Laurent Roten, Nicolas Rodondi, Moa Haller, Andreas S. Müller, Alain M. Bernheim, Jürg H. Beer, Giorgio Moschovitis, Maria Luisa De Perna, David Conen, Stefan Osswald, Christian Sticherling, Philip Haaf, Philipp Krisai, Michael Kühne, Christine S. Zuern

**Affiliations:** 1 Department of Cardiology, University Hospital Basel, Basel, Switzerland; 2 Cardiovascular Research Institute Basel, University Hospital Basel, Basel, Switzerland; 3 Department of Clinical Research, University of Basel and University Hospital Basel, Basel, Switzerland; 4 Department of Cardiology, Inselspital, Bern University Hospital, University of Bern, Bern, Switzerland; 5 Institute of Primary Health Care, University of Bern, Bern, Switzerland; 6 Department of General Internal Medicine, Inselspital, Bern University Hospital, University of Bern, Bern, Switzerland; 7 Department of Cardiology, Triemli Hospital Zürich, Zürich, Switzerland; 8 Department of Medicine, Cantonal Hospital of Baden and Molecular Cardiology, University Hospital of Zurich, Zurich, Switzerland; 9 Division of Cardiology, Ente Ospedaliero Cantonale, Cardiocentro Ticino Institute, Regional Hospital of Lugano, Lugano, Switzerland; 10 Population Health Research Institute, McMaster University, 237 Barton Street East, Hamilton, Ontario, Canada

**Keywords:** Atrial fibrillation, Atrial fibrillation burden, Cardiac magnetic resonance imaging, Discrimination, Left atrial remodeling, Risk stratification

## Abstract

**Background:**

Recent studies indicate that atrial fibrillation (AF) burden has prognostic implications.

**Objective:**

We aimed to assess the ability of clinical and cardiac imaging variables to stratify between high and low AF burden.

**Method:**

Data from the prospective, multicenter Swiss-AF Burden study were analyzed. Patients underwent a 7-day Holter electrocardiogram and native cardiac magnetic resonance imaging. AF burden, defined as the percentage of time in AF during the 7-day Holter electrocardiogram, was dichotomized into low (<10%) or high (≥10%). Logistic regression models were built, and discriminative performance was evaluated by comparing the area under the curve (AUC).

**Results:**

A total of 170 patients were enrolled (median age 72 years; 18% female); 26% (n = 44) had high AF burden. Variables selected for the clinical model were age (odds ratio 1.09; 95% confidence interval 0.40–2.74), male sex (1.05; 1.00–1.11), and body mass index (1.14; 1.06–1.24). The imaging model included left atrial maximal volume index (1.04; 1.01–1.06), left ventricular end-diastolic volume index (0.93; 0.90–0.96), right atrial fractional area change (0.94; 0.89–0.98), and left ventricular ejection fraction (0.87; 0.81–0.94). The AUCs for the clinical and imaging models were 0.67 (0.58–0.77) and 0.91 (0.84–0.98), respectively. Combining both models yielded an AUC of 0.92 (0.86–0.99), with no substantial improvement over the imaging model alone.

**Conclusion:**

Cardiac imaging variables clearly outperformed clinical variables in their ability to stratify between high and low AF burden, suggesting their potential as a tool for estimating AF burden.

## Graphical abstract

**Figure:**
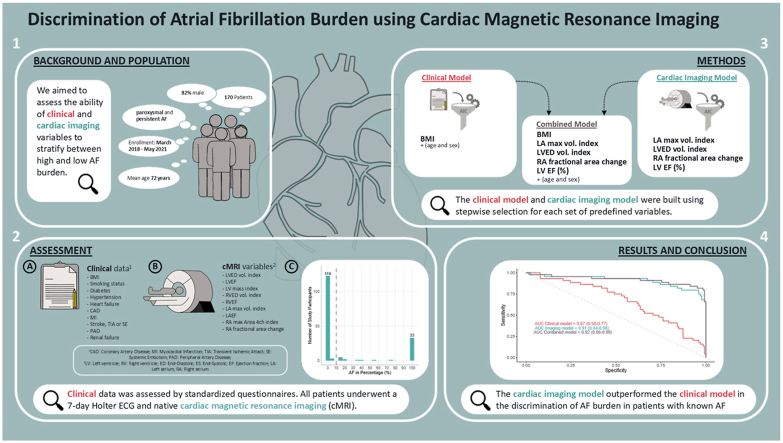

**Key Findings**

▪ In a contemporary cohort of patients with atrial fibrillation (AF), only 1 in 4 patients exhibited a high AF burden (>10%) during 7-day Holter monitoring.
▪ A multivariable model based solely on clinical variables demonstrated only moderate discrimination between patients with high and low AF burden.
▪ Cardiac imaging parameters—including left atrial maximum volume index, left ventricular (LV) end-diastolic volume index, right atrial fractional area change, and LV ejection fraction—substantially improved the discriminatory performance for AF burden, achieving an area under the curve of >0.90.
▪ The combination of clinical and imaging variables did not further enhance the ability to discriminate between high and low AF burden compared with imaging parameters alone.

## Introduction

Atrial fibrillation (AF) is traditionally classified into paroxysmal, persistent, or permanent.^1^ However, this categorization is most likely insufficient to capture the complexity of the disease.^2^ AF burden is defined as the longest AF episode, the number of AF episodes, or the total duration of all AF episodes within a measured time (expressed as a percentage).^3^ AF burden has been shown to be an independent risk factor for adverse outcomes^4^ such as stroke,^5^^,^^6^ even after adjustment for risk scores such as the ATRIA stroke risk score or CHA_2_DS_2_-VASc score.^7^ A novel risk model including AF burden together with the CHA_2_DS_2_-VASc score may provide a more precise classification of stroke risk than the CHA_2_DS_2_-VASc score alone, which may particularly improve the assessment and management of intermediate-risk patients.^8^^,^^9^

Increasing insights into AF burden are primarily attributable to advancements in electrocardiogram (ECG) software and technology, given that there now exist various easy-to-install continuous AF monitoring devices, such as implantable cardiac monitors, wearables, and ECG patches.

In an aging population, the number of patients experiencing AF is increasing. Available tools to measure AF burden may be cost-intensive, and results need to be validated by clinicians. Consequently, it is not feasible to assess AF burden in all patients with AF owing to the scarcity of resources and rising health care costs.^10^^,^^11^ It remains uncertain which criteria should guide the decision to further investigate AF burden. Although clinical and imaging risk factors for AF progression and recurrence are well described in the literature,^12^^,^^13^ the potential of these variables to estimate AF burden remains unexplored.

Our study aimed to determine the performance of clinical variables, imaging variables, and their combination in discrimination between high and low AF burden in a patient population with known AF.

## Methods

### Study design and patient population

**Supplemental Figure 1:**
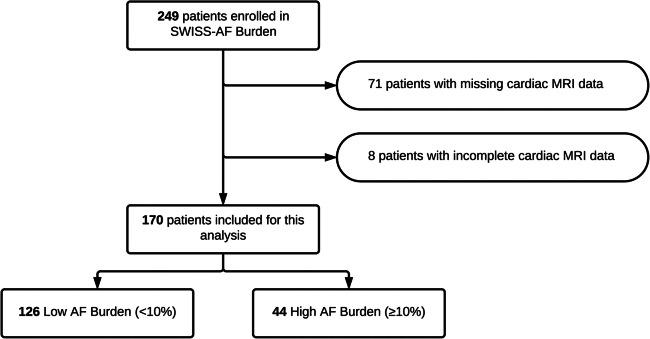


This cross-sectional analysis (ClinicalTrials.gov identifier: NCT05389228) included patients enrolled in the Swiss-AF Burden study. The Swiss-AF Burden study is nested within the Swiss-AF cohort (Swiss Atrial Fibrillation Cohort study), a prospective, multicenter observational study in Switzerland. The primary goal of the Swiss-AF Burden study was to investigate AF burden and its association with adverse outcomes. Recruitment for this study was conducted between March 2018 and May 2021. Patients with paroxysmal or persistent AF enrolled in Swiss-AF were eligible for participation in the Swiss-AF Burden study. AF type classification was in accordance with the 2010 AF guidelines of the European Society of Cardiology. Patients who were unwilling or unable to perform a 7-day Holter ECG monitoring or a cardiac magnetic resonance imaging (cMRI) (eg, owing to claustrophobia or a cardiac device) were excluded from the study. A total of 249 patients were enrolled in the Swiss-AF Burden study (Supplemental Figure 1). Owing to restrictions and data collection challenges caused by the coronavirus disease 2019 pandemic, 71 patients had missing cMRI data and were consequently excluded. In addition, 8 patients were excluded because of incomplete cMRI data. Consequently, the final analysis set comprised 170 patients.

An informed consent was signed by all study participants. The study protocol was approved by the responsible ethics commission and complied with the Helsinki declaration.

### Assessment of AF burden

AF was measured using a validated 2-lead Holter ECG (Lifecard CF, Spacelabs Healthcare, distributed by Leuag AG, Switzerland) over a 7-day period (median 167 hours; interquartile range 6 hours). 12 of 170 Holter recordings (7%) were terminated prematurely.

AF episodes were detected with the help of an automated algorithm using the SRAclinic software (Apoplex Medical Technologies, Pirmasens, Germany). The algorithm assessed QRS complexes of the ECG data and classified them as supraventricular or ventricular. To ensure accurate and consistent measurement of AF duration across the study cohort, all Holter recordings were reviewed manually by 2 independent physicians of the study team. In case of significant discrepancies (>5%) between analyses, a third expert was consulted. Details of the manual AF burden assessment have been described previously.^14^

**Figure 1:**
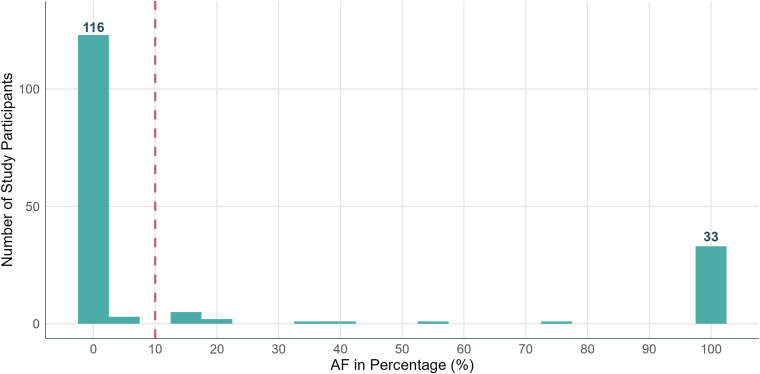
Distribution of AF in % (time in AF / total recording time). Distribution of AF burden of the 170 patients included in this study. AF burden in % is defined as the total time in AF divided by the recorded time of a 7-day period using Holter electrocardiogram monitoring. The *red dotted line* indicates the 10% cutoff value. AF = atrial fibrillation.

We defined AF burden as the total duration of all AF episodes divided by the total recording time and expressed as a percentage. Owing to the distribution polarity of AF burden (Figure 1), we decided to use AF burden as a dichotomized variable. In accordance with the results of the KP-RHYTHM study,^7^ we divided the AF burden into 2 groups: low (<10%) and high (≥10%) AF burden.

### Clinical data

Clinical data were collected in the context of the ongoing Swiss-AF study. The data were assessed using standardized questionnaires. Items were collected on patients’ lifestyle, medication, and medical history. Smoking status was categorized into current smokers and nonsmokers. The body mass index (BMI) was calculated as weight (kg) divided by height (m^2^). The body surface area was calculated as weight (kg) multiplied by height (cm) divided by 3600. The clinical data closest to the date of Holter monitoring (median 24 days; interquartile range 69 days) were used for the analysis.

### cMRI

Imaging data were acquired close to the time of the clinical visit, with the cMRI performed preferably before or near the 7-day Holter monitoring period.

The cMRI scans were performed using a dedicated 1.5T or 3T MRI scanner, equipped with multichannel phased-array surface receiver coils capable of accommodating up to 32 channels. Contrast agents were not required for these scans. The scanning protocol lasted approximately 15 minutes and included several sequences.

Initially, multiplanar scout images were obtained to plan the 2-dimensional imaging planes. Functional imaging of the cardiac chambers, specifically the left (LV) and right ventricles and the left (LA) and right atria (RA), was performed using cine white-blood balanced steady-state free precession sequences in both long- and short-axis planes.

The sequences were acquired with a matrix size of 224 × 198, a field of view of 34 × 30 cm, a repetition time of 2.8 ms, an echo time of 1.2 ms, and a flip angle of 57 degrees. The in-plane resolution was 1.5 × 1.5 mm^2^, with a slice thickness of 6 mm for long-axis images and 8 mm for short-axis images. The temporal resolution was set to 25 frames per cardiac cycle.

### Statistical analysis

Baseline characteristics are presented overall and stratified by AF burden (high vs low AF burden). Continuous variables are presented as mean ± standard deviation or median (first quartile, third quartile) and are compared using the *t* test or the Wilcoxon test if strongly skewed. Categorical variables are presented as numbers (percentages) and compared using χ^2^ tests.

#### Variable selection for discrimination models

**Supplemental Figure 2:**
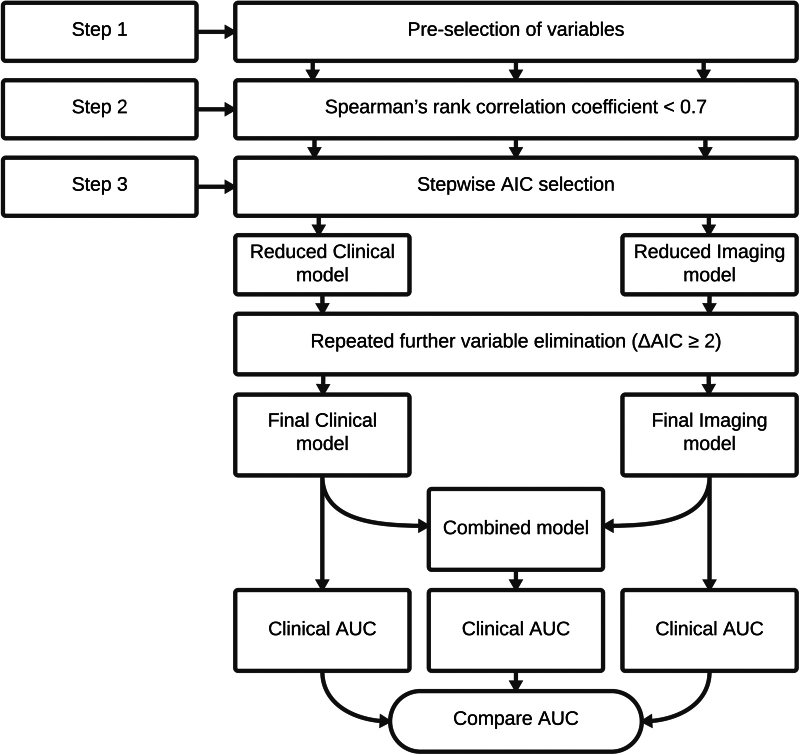


Owing to the relatively small sample size and limited number of cases with high AF burden compared with the large pool of potential independent variables, we decided to follow a 3-step approach for variable selection in our models (Supplemental Figure 2):

• Step 1: clinical variables were selected after a thorough literature review and their obtainability in a clinical setting. All cMRI variables related to volume, mass, and function were included in the analysis. Measurements were indexed by body surface area (in m^2^) to facilitate comparison between study patients.
• Step 2: separate correlation analyses were performed for clinical and cMRI variables. Variables demonstrating a high correlation (Spearman’s rank correlation coefficient ≥0.7) were excluded. End-diastolic measures were retained over end-systolic measures, and LA maximal volume (LA max vol.) was preferred over LA minimum volume (LA min vol.).
• Step 3: we constructed 2 full models, 1 incorporating all clinical and another with all cardiac imaging variables. A stepwise elimination approach using the Akaike information criterion (AIC) was applied to each full model, resulting in optimized, reduced versions.

To try to reduce the models as much as possible, we further excluded variables that, when removed from the model, did not induce an increase in AIC (ΔAIC) of more than 2 units.^15^ This approach allowed for focusing on the most influential variables while maintaining model performance.

#### Multiple logistic regression and discriminative performance

Multiple logistic regression analysis was performed to develop models for discriminating AF burden (high vs low):

I) Clinical model: a multivariable model including clinical variables, adjusted for sex and age
II) Cardiac imaging model: a multivariable model including cardiac imaging variables
III) Combined clinical and imaging model: a multivariable model including all selected clinical and imaging variables, adjusted for sex and age

To assess and compare the discriminatory power of the clinical, cardiac imaging, and combined models, receiver operating characteristic curves were generated for each model, and the area under the curve (AUC) was computed. To statistically evaluate the differences between 2 AUC values, the DeLong test was applied for non-nested models. We compared the fit of nested models using the likelihood ratio test.

#### Internal validation: Bootstrap analysis

Furthermore, we assessed the overfitting of the combined model using bootstrap analysis with 3000 repetitions. The variable selection process was conducted for each bootstrap sample to generate a corresponding model. We calculated the difference between the bootstrapped model and the original model’s AUC as a measure of bias. We used the mean bias to calculate a bias-corrected AUC.

All analyses were performed using R 4.2.2 (R Core Team [2021], R Foundation for Statistical Computing, Vienna, Austria).

## Results

### Baseline characteristics

In our population, 116 patients (68.2%) had an AF burden of 0%, 33 patients (19.4%) 100%, and 21 patients (12.4%) between 0% and 100% (Figure 1). The study population was predominantly male (82%); the median age of the participants was 72 years. Baseline characteristics stratified by high and low AF burden (cutoff 10%) are presented in Table 1. Overall, 126 patients (74%) had a low AF burden, whereas a high AF burden was present in 44 patients (26%). Patients with high AF burden had a higher BMI (28 vs 26; *P* = .002) than patients with low AF burden.

**Table 1:** Baseline characteristics stratified by AF burden (cutoff 10%)

Population characteristics	Overall, N = 170∗		AF burden (%)	
		<10%, n = 126 (74%)∗	≥10%, n = 44 (26%)∗	*P* value†
Sex				.7
Female	31 (18)	22 (17)	9 (20)	
Age (y)	72 [69–77]	72 [69–77]	74 [70–82]	.14
AF type				<.001‡
Paroxysmal	103 (61)	88 (70)	15 (34)	
Persistent (>7 d, ECV)	67 (39)	38 (30)	29 (66)	
CHA_2_DS_2_-VASc score	3 [2–4]	3 [2–4]	4 [2–4]	.14
Body mass index (kg/m^2^)	26 [24–30]	26 [23–28]	28 [25–32]	.003‡
Current smoker	12 (7)	9 (7)	3 (7)	>.9
History of diabetes	17 (10)	11 (9)	6 (14)	.4
History of hypertension	100 (59)	70 (56)	30 (68)	.14
History of heart failure	27 (16)	16 (13)	11 (25)	.055
History of CAD	43 (25)	29 (23)	14 (32)	.2
History of MI	16 (9)	10 (8)	6 (14)	.4
History of stroke, TIA, or SE	40 (24)	32 (25)	8 (18)	.3
History of PAD	8 (5)	6 (5)	2 (5)	>.9
History of renal failure	28 (16)	21 (17)	7 (16)	>.9
AAD therapy	31 (18)	22 (17)	9 (20)	.7
Previous PVI	57 (34)	49 (39)	8 (18)	.012‡
Previous ECV	60 (37)	41 (34)	19 (45)	.2
LVED vol. index (mL/m^2^)	76 [65–87]	80 [68–88]	67 [57–77]	<.001‡
LVES vol. index (mL/m^2^)	34 [27–40]	33 [27–40]	35 [30–41]	.2
LVEF (%)	56 [48–61]	57 [52–63]	46 [41–53]	<.001‡
LV mass index (g/m^2^)	65 [58–74]	67 [59–75]	62 [52–67]	.002‡
RVED vol. index (mL/m^2^)	78 [67–89]	80 [70–92]	71 [60–83]	.001‡
RVES vol. index (mL/m^2^)	37 [29–47]	38 [29–46]	37 [32–49]	.3
RVEF (%)	52 [43–58]	54 [47–60]	42 [36–49]	<.001‡
LA max vol. index (mL/m^2^)	50 [40–64]	48 [36–57]	60 [49–71]	<.001‡
LA min vol. index (mL/m^2^)	28 [20–45]	25 [17–36]	49 [37–58]	<.001‡
LAEF (%)	41 [24–54]	47 [36–56]	22 [14–28]	<.001‡
RA max area 4ch index (cm^2^/m^2^)	12 [10–14]	12 [10–14]	14 [11–17]	.009‡
RA min area 4ch index (cm^2^/m^2^)	9 [7–11]	8 [6–10]	11 [9–15]	<.001‡
RA fractional area change (%)	31 [20–39]	33 [28–42]	11 [6–22]	<.001‡

AAD = antiarrhythmic drug; AF = atrial fibrillation; CAD = coronary artery disease; CHA2DS2-VASc = congestive heart failure, hypertension, age ≥75 (2 points), diabetes mellitus, previous stroke/TIA/thromboembolism (2 points), vascular disease, age 65–74, female sex; ECV = electrocardioversion; LA = left atrium; LAEF = left atrial ejection fraction; LV = left ventricle; LVED = left ventricular end-diastolic; LVEF = left ventricular ejection fraction; LVES = left ventricular end-systolic; max = maximal; MI = myocardial infarction; min = minimum; PAD = peripheral artery disease; PVI = pulmonary vein isolation; RA = right atrium; RVED = right ventricular end-diastolic; RVEF = right ventricular ejection fraction; RVES = right ventricular end-systolic; SE = systemic embolism; TIA = transient ischemic attack; vol. = volume.

∗ n (%); median [interquartile range].

† Pearson’s χ^2^ test; Wilcoxon rank sum test; Fisher’s exact test.

‡ Statistically significant.

In contrast to clinical characteristics, most cMRI parameters demonstrated significant differences between patients with low and high AF burden, as presented in Table 1. The LV end-diastolic volume index (LVEDVI) and the LV ejection fraction (LVEF) were lower in the high AF burden group (80 mL/m^2^ vs 67 mL/m^2^ [*P* < .001] and 57% vs 46% [*P* < .001]), whereas the LA max vol. index was higher in the high AF burden group (60 mL/m^2^ vs 48 mL/m^2^; *P* < .001). The RA fractional area change was lower in the high AF burden group (33% vs 11%; *P* < .001).

### Variable selection

All clinical and cMRI variables examined in the study are presented in Tables 2 and 3. The tables provide a step-by-step visualization, moving from left to right, to illustrate which variables were included and which were excluded at each stage. Among the clinical variables, only BMI emerged as an essential variable for model fit (Table 2). Among the imaging variables, LVEF (%), LVEDVI (mL/m^2^), LA max vol. index (mL/m^2^), and RA fractional area change (%) contributed to the model fit (Table 3).

**Table 2:** Clinical Model for discrimination of AF burden

Variable	Full model	Stepwise selection	Reduced model (ΔAIC)	Final model (ΔAIC)
Sex	**✓**	**✓**	**✓**	**✓**
Age (y)	**✓**	**✓**	**✓**	**✓**
Body mass index (kg/m^2^)	**✓**	**✓**	**✓** (10.89)	**✓** (10.87)
Current smoker	**✓**	✗	**-**	**-**
History of diabetes	**✓**	✗	**-**	**-**
History of hypertension	**✓**	✗	**-**	**-**
History of heart failure	**✓**	**✓**	✗ (0.63)	**-**
History of CAD	**✓**	✗	**-**	**-**
History of stroke, TIA, or SE	**✓**	✗	**-**	**-**
History of PAD	**✓**	✗	**-**	**-**
History of MI	**✓**	✗	**-**	**-**
AIC	199.39	186.83	187.46	187.46

Step-by-step visualization from left to right illustrating the creation of the final model based on automated stepwise selection and further comparison with reduced models with 1 variable removed, calculating the ΔAIC. A ΔAIC of ≥2 indicates a meaningful change in model performance. In the final model, no variable can be removed without resulting in a ΔAIC of ≥2. The clinical model remains adjusted for age and sex.

**✓** = included variable; ✗ = excluded variable; AF = atrial fibrillation; AIC = Akaike information criterion; CAD = coronary artery disease; MI = myocardial infarction; PAD = peripheral artery disease; TIA = transient ischemic attack; SE = systemic embolism.

**Table 3:** Cardiac Imaging Model for discrimination of AF burden

Variable	Full model	Stepwise reduced	Reduced model (ΔAIC)		Final model (ΔAIC)
LVED vol. index (mL/m^2^)	**✓**	**✓**	**✓** (6.48)	**✓** (9.09)	**✓** (22.33)
LVEF (%)	**✓**	**✓**	**✓** (8.32)	**✓** (13.63)	**✓** (14.60)
LV mass index (g/m^2^)	**✓**	**✓**	**✓** (0.85)	✗ (0.56)	**-**
RVEF (%)	**✓**	✗	**-**	**-**	**-**
RVED vol. index (mL/m^2^)	**✓**	✗	**-**	**-**	**-**
LA max vol. index (mL/m^2^)	**✓**	**✓**	**✓** (3.62)	**✓** (6.56)	**✓** (7.6)
LAEF (%)	**✓**	**✓**	✗ (0.19)	**-**	**-**
RA max area 4ch index (cm^2^/m^2^)	**✓**	✗	**-**	**-**	**-**
RA fractional area change (%)	**✓**	**✓**	**✓** (4.31)	**✓** (6.92)	**✓** (6.59)
AIC	112.1	107.35	107.6	108.12	108.12

Step-by-step visualization from left to right illustrating the creation of the final model based on automated stepwise selection and further comparison with reduced models with 1 variable removed, calculating the ΔAIC. A ΔAIC of ≥2 indicates a meaningful change in model performance. In the final model, no variable can be removed without resulting in a ΔAIC of ≥2.

**✓** = included variable; ✗ = excluded variable; AF = atrial fibrillation; AIC = Akaike information criterion; LV = left ventricle; LA = left atrium; LAEF = left atrial ejection fraction; LVED = left ventricular end-diastolic; LVEF = left ventricular ejection fraction; max = maximal; RA = right atrium; RV = right ventricle; RVED = right ventricular end-diastolic; RVEF = right ventricular ejection fraction; vol. = volume.

### Comparison of model discriminatory power

**Figure 2:**
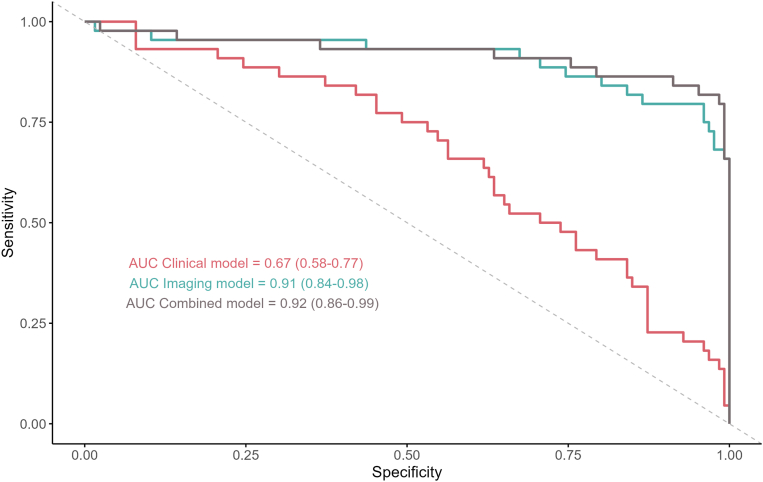
ROC curves of the clinical, imaging, and combined model (AF cutoff 10%). ROC curves for the clinical, imaging, and combined models at AF burden cutoffs of 10%. The clinical and combined models were adjusted for age and sex. Model performance is expressed as the AUC with 95% confidence intervals. AF = atrial fibrillation; AUC = area under the curve; ROC = receiver operating characteristic.

The receiver operating characteristic curves for all models are presented in Figure 2. The clinical model showed an AUC of 0.67 (95% confidence interval [CI] 0.58–0.77), indicating a moderate discriminative ability. In comparison, the imaging model had a higher AUC of 0.91 (95% CI 0.84–0.98), indicating superior discriminatory ability (Delong test, *P* < .005). The combined model using both clinical and imaging data achieved the highest AUC of 0.92 (95% CI 0.86–0.99), demonstrating the best discriminatory ability among the 3 models. The difference in AUC between the cardiac imaging model and the combined model was not substantial, although there is some evidence for a better fit of the combined model (likelihood ratio test, *P* = .037). Bootstrapping analysis with 3000 iterations demonstrates a mean bias of −0.03 CI (95% CI −0.07 to 0.02) with a bias-corrected AUC of 0.89 for the combined model.

### Comparison of the variable effects across the models

**Figure 3:**
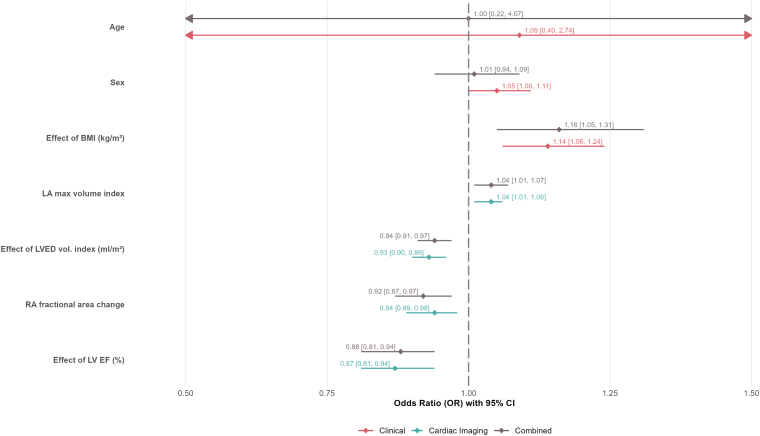
Forrest plot of the clinical, imaging, and combined model. Association between clinical and imaging variables and high AF burden using logistic regression analysis. The clinical and combined models were adjusted for age and sex. All variables are log-transformed and standardized. The model is sorted by OR = exp (β-coefficient). AF = atrial fibrillation; BMI = body mass index; CI = confidence interval; LA = left atrial; LVED vol. = left ventricular end-diastolic volume; LVEF = left ventricular ejection fraction; OR = odds ratio; RA = right atrium.

Figure 3 illustrates and compares the estimated effects of variables (odds ratio [OR]; 95% CI) on AF burden across the 3 models: clinical, cardiac imaging, and combined (CIs not corrected for variable selection). Age had minimal impact, with wide CIs, and the effect of sex in the clinical model (OR 1.05; CI 1.00–1.11) diminished when imaging variables were included. A higher BMI increased the odds of AF burden in the clinical model (OR 1.14; CI 1.06–1.24), with little change in the combined model. Cardiac imaging variables remained consistent across models. The LVEDVI (OR 0.93; CI 0.90–0.96), RA fractional area change (OR 0.94; CI 0.89–0.98), and LVEF (OR 0.87; CI 0.81–0.94) correlated with a lower AF burden. Conversely, a larger LA max vol. index (OR 1.04; CI 1.01–1.06) indicated an increase in AF burden.

## Discussion

In this cross-sectional analysis, 170 patients with AF underwent a 7-day Holter ECG, clinical assessment using a questionnaire, and cMRI to identify clinical and imaging characteristics that could help physicians distinguish between patients with a high and low AF burden.

This study has generated several important findings. First, in a contemporary cohort of patients with AF, only 1 in 4 patients had a high AF burden of >10%. Second, a multivariable model using clinical variables had only a moderate ability to discriminate between high and low AF burden. Third, cardiac imaging measurements, including LA max vol. index, LVEDVI, RA fractional area change, and LVEF, showed a substantially improved discriminatory ability regarding AF burden with an AUC of >90%. Finally, combining clinical and imaging variables did not further improve the ability to discriminate between high and low AF burden.

### Distribution of AF burden and AF pattern

In our population, many patients were either constantly in AF or had no AF episodes during the 7-day Holter monitoring period. Only 12% had an AF burden between 0% and 100%. Several reasons could explain this distribution.

First, a 7-day monitoring period might be insufficient to accurately capture the full AF burden given that intermittent monitoring has been demonstrated to miss the detection of newly diagnosed AF^16^ or AF recurrence in patients with paroxysmal AF.^17^ In a recent consensus document,^18^ continuous rhythm monitoring for a minimum of 28 days was proposed as the gold standard for the assessment of a “true AF burden.” AF burden might have been either underestimated or overestimated in our population given that a previous study comparing various simulated intermittent monitoring strategies in patients with implantable loop recorders has demonstrated variability in AF burden estimation.^19^ In contrast, the use of a 7-day Holter ECG is more common in a clinical setting, and compared with an implantable loop recorder, it is less expensive and noninvasive.

Second, a relevant proportion of patients in our cohort received rhythm control, including antiarrhythmic drugs, electrical cardioversion, and pulmonary vein isolation. These therapies contributed to the reduction in AF burden.^20^ Third, the distribution of AF burden may give us an insight into the nature of AF. It is possible that AF is not a linearly progressive disease, but rather more dichotomous in nature, with a relatively short transitional phase, as also proposed by Steinberg et al.^21^ In their analysis of 3 independent cohorts, AF burden clustered predominantly at the extremes, resulting in a bimodal distribution. This distribution might reflect the underlying pathophysiology of AF, in which persistent or permanent forms are associated with advanced atrial cardiomyopathy and high arrhythmic load.

### Dichotomization of AF burden

**Supplemental Figure 3:**
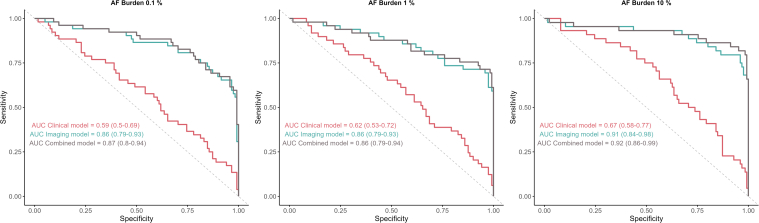


Although AF burden would ideally be analyzed as a continuous measure, pronounced polarization within our cohort necessitated dichotomization, despite the lack of an empirically established threshold for predicting clinical outcomes.^3^ The observed correlation between increasing AF burden and adverse clinical outcomes even suggests a possible proportional relationship,^4^ which raises the question of whether such a threshold could be defined at all, especially because it may differ depending on the chosen outcome. Nonetheless, AF burden could be used as a metric helpful in the guidance of antiarrhythmic therapy, given that studies suggest that a reduction in AF burden leads to better clinical outcome^22^ and quality of life.^23^ In the present analysis, we adopted a cutoff based on findings from the KP-RHYTHM study^7^ showing that an AF burden of ≥11.4% (the highest tertile in their study) was associated with a significantly higher stroke rate. An additional analysis using alternative AF burden cutoffs (0.1% and 1%) is presented in Supplemental Figure 3; the overall findings and the main conclusions were consistent across all evaluated thresholds.

### Clinical variables distinguishing AF burden

BMI was identified as the most important clinical variable. This is in line with other studies, showing that obesity is associated with the incidence,^24^ progression,^25^ and higher recurrence^26^ of AF. Variables included in the CHA_2_DS_2_-VASc score, such as a history of hypertension^27^ or heart failure,^28^ are associated with an increased likelihood of AF progression. However, after unbiased statistical variable selection, these variables were not retained in the clinical model and therefore did not play a relevant role in effectively distinguishing patients with high vs low AF burden. If AF burden turns out to be a variable independent of the CHA_2_DS_2_-VASc score, the concept of a CHA_2_DS_2_-VASc burden score^9^ may become clinically relevant. However, whether AF burden and the CHA_2_DS_2_-VASc score truly do not interact with each other needs to be further elucidated.

### Imaging variables distinguishing AF burden

Imaging variables showed significant differences between the 2 AF burden groups at baseline. Using the AIC method to narrow variables, 2 were integrated into the imaging model: LVEDVI and LVEF. Lower LVEDVI was associated with a higher AF burden. This may potentially be attributed to LV stiffness and reduced compliance,^29^ resulting in impairment of LV filling. The lower LVEF observed in the high AF burden group could be related to loss of atrial systole and irregular ventricular beats.^30^ As a marker of systolic dysfunction, LVEF is closely related to AF, and various studies have shown that AF can be both a cause and a consequence of heart failure.^31^

LA max vol. index and LA ejection fraction were incorporated into the cardiac imaging model using an automated stepwise selection approach. Both atrial parameters are considered established markers of underlying atrial cardiomyopathy, a pathophysiologically highly relevant entity characterized by structural remodeling, fibrosis, and impaired atrial compliance. Atrial cardiomyopathy increases vulnerability to AF, the progression of which may, in turn, be further promoted by AF itself.^32^^,^^33^ However, when being more stringent in model reduction, LA ejection fraction was excluded from the model. This might not apply to other populations, given that studies demonstrated that LA dysfunction is already present in patients with paroxysmal AF compared with those with sinus rhythm, regardless of LA size.^34^^,^^35^

Similarly, the RA fractional area change, an indicator of RA dysfunction, was reduced in the high AF burden group. This suggests that the RA, like the LA, may undergo remodeling^36^ and impaired function in patients with AF.^37^ Although RA alterations seem to be less relevant for clinical outcomes compared with LA changes,^38^^,^^39^ they may nevertheless remain of interest as a parameter for estimating AF burden.

Although the inclusion of the selected clinical variables in the combined model improved model fit compared with the cardiac imaging alone model, the model’s performance in discriminating AF burden was not substantially enhanced. This implies that imaging variables may play a predominant role in the assessment of AF burden.

### Strengths and limitations

This analysis is among the first studies to evaluate AF burden and its clinical and imaging predictors in detail in a well-characterized contemporary cohort, using long-term ECG monitoring over a 7-day period. Some limitations should be considered when interpreting the results. First, more accurate AF burden data could have been achieved by a longer monitoring period, as proposed in the consensus document. Therefore, we provide an estimate of AF burden rather than a definitive quantification. However, 7-day Holter monitoring is common in clinical routine. Second, the dichotomization of AF burden with a cutoff of 10% remains arbitrary, given that different cutoffs are proposed in the literature. Third, owing to the study design, several clinical variables of interest, such as relevant blood biomarkers and ECG abnormalities, could not be included in the clinical model, likely limiting its strength and discriminatory performance. Fourth, antiarrhythmic therapy may have influenced AF burden without altering the MRI-derived parameters that were correlated with burden. Fourth, we cannot judge whether echocardiographically derived parameters would have resulted in equal results. However, CMR is considered the standard tool for volumetry and is less operator dependent than echocardiography. Finally, owing to the study’s inclusion criteria, the generalizability of our findings to other AF populations may be limited.

## Conclusion

In our analysis, we were able to show that precise measurements of cardiac volumetry and function can identify patients with a high AF burden. This might potentially help clinicians in selecting future monitoring strategies for patients with AF. Although growing evidence suggests that higher AF burden is associated with adverse clinical outcomes, definitive thresholds and causal relationships remain uncertain and should be clarified in future prospective studies.

## Disclosures

C. Sticherling is a member of Medtronic Advisory Board Europe and Boston Scientific Advisory Board Europe and received educational grants from Biosense Webster and Biotronik, a research grant from the European Union’s FP7 program and Biosense Webster, and lecture and consulting fees from Abbott, Medtronic, Biosense Webster, Boston Scientific, MicroPort, and Biotronik, all outside the submitted work. C.S. Zuern reports a research grant from Freiwillige Akademische Gesellschaft Basel and a travel grant from Cardiomatics. D. Conen received consultancy fees from Trimedics and speaker fees from Servier. G. Moschovitis has received consultant fees for taking part in advisory boards from Novartis and AstraZeneca outside of the submitted work. M. Kühne reports grants from the Swiss National Science Foundation (grant numbers 33CS30_148474, 33CS30_177520, 32473B_176178, and 32003B_197524), the Swiss Heart Foundation, the Foundation for Cardiovascular Research Basel, the University of Basel, Bayer, Bristol Myers Squibb (BMS), Boston Scientific, Daiichi Sankyo, and Pfizer; personal fees from Abbott, Boston Scientific, and Daiichi Sankyo; and royalties from Springer Nature. N. Rodondi received a grant from the Swiss Heart Foundation. P. Krisai reports speaker fees from BMS/Pfizer and grants from the Swiss National Science Foundation, the Swiss Heart Foundation, the Foundation for Cardiovascular Research Basel, and the Machaon Foundation. S. Aeschbacher received a speaker fee from Roche Diagnostics. S. Osswald received research grants from the Swiss National Science Foundation, the Swiss Heart Foundation, the Foundation for Cardiovascular Research Basel, and Roche and educational and speaker office grants from Roche, Bayer, Novartis, Sanofi, AstraZeneca, Daiichi Sankyo, and Pfizer. L. Roten received research grants from Medtronic, the Swiss National Foundation, the Swiss Heart Foundation, the Immanuel and Ilse Straub Foundation, and the Sitem Insel Support Fund, all for work outside the submitted study. He received speaker fees/honoraria from Biosense Webster, Boston Scientific, Abbott, and Medtronic. T. Reichlin received research grants from the Swiss National Science Foundation and the Swiss Heart Foundation, all for work outside the submitted study; speaker/consulting honoraria or travel support from Abbott/St. Jude Medical, AstraZeneca, Brahms, Bayer, Biosense Webster, Biotronik, Boston Scientific, Daiichi Sankyo, Medtronic, Pfizer-BMS, and Roche, all for work outside the submitted study; and support for his institution’s fellowship program from Abbott/St. Jude Medical, Biosense Webster, Biotronik, Boston Scientific, and Medtronic for work outside the submitted study. The remaining authors have no disclosures to report.

## Footnotes

Appendix Supplementary data associated with this article can be found in the online version at https://doi.org/10.1016/j.hroo.2026.03.011.
