# Supplementary material for: Discrimination of atrial fibrillation burden using cardiac magnetic resonance imaging
Source: Heart Rhythm O2. 2026 Mar 19;7(6):1095–104. doi: 10.1016/j.hroo.2026.03.011 (PMC13307481; doi:10.1016/j.hroo.2026.03.011)
Supplement: Supplemental Material [file mmc1.docx]

**Supplementary**

**Discrimination of Atrial Fibrillation Burden using**

**Cardiac Magnetic Resonance Imaging**

Andreas U. Gasser MD^1,2^, Stefanie Aeschbacher PhD^1,2^, Michael Coslovsky PhD^1,3^,

Vincent Meier MD^1,2^, Tanja Ruoff BMed^2^, Tobias Reichlin MD^4^, Laurent Roten MD^4^, Nicolas Rodondi MD^5,6^, Moa Haller MD^5^, Andreas S. Müller MD^7^, Alain M. Bernheim MD^7^, Jürg H. Beer MD^7,8^, Giorgio Moschovitis MD^9^, Maria Luisa De Perna MD^9^, David Conen MD MPH^10^, Stefan Osswald MD^1,2^, Christian Sticherling MD^1,2^, Philip Haaf MD^1,2^, Philipp Krisai MD^1,2^, Michael Kühne MD*^1,2^, Christine S. Zuern MD*^1,2^ for the Swiss-AF Investigators

*^1^ Department of Cardiology, University Hospital Basel, Basel, Switzerland*

*^2^ Cardiovascular Research Institute Basel, University Hospital Basel, Basel, Switzerland*

*^3^ Department of Clinical Research, University of Basel and University Hospital Basel, Basel, Switzerland*

*^4^ Department of Cardiology, Inselspital, Bern University Hospital, University of Bern, Bern, Switzerland*

*^5^ Institute of Primary Health Care (BIHAM), University of Bern, Bern, Switzerland*

*^6^ Department of General Internal Medicine, Inselspital, Bern University Hospital, University of Bern, Bern, Switzerland*

*^7^ Department of Cardiology, Triemli Hospital Zürich, Zürich, Switzerland*

*^8^ Department of Medicine, Cantonal Hospital of Baden and Molecular Cardiology, University Hospital of Zurich, Zurich, Switzerland*

*^9^ Division of Cardiology, Ente Ospedaliero Cantonale (EOC), Cardiocentro Ticino Institute, Regional Hospital of Lugano, Lugano, Switzerland*

*^10^ Population Health Research Institute, McMaster University, 237 Barton Street East, Hamilton, ON, Canada*

**Shared last authors*

**Short title:** Swiss AF Burden

**Funding:** Swiss Heart Foundation, University of Basel, Freiwillige Akademische Gesellschaft Basel, Swiss National Science Foundation (grant numbers 33CS30_148474, 33CS30_177520, 32473B_176178, and 32003B_197524)

**Keywords:** Atrial fibrillation, atrial fibrillation burden, cardiac magnetic resonance imaging, discrimination, left atrial remodeling, risk stratification

**Corresponding author:**

Christine Meyer-Zürn, MD FESC

Department of Cardiology, University Hospital Basel

Petersgraben 4, CH-4031 Basel, Switzerland

Email: [christine.meyerzuern@usb.ch](mailto:christine.meyerzuern@usb.ch)


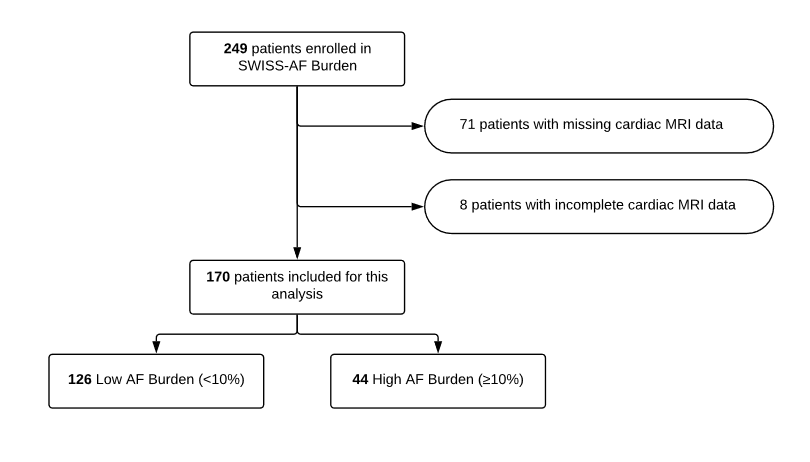
 **Supplementary Figure 1: Study design and patient population**

*Flowchart of patient inclusion in the SWISS-AF Burden study. A total of 249 patients were initially enrolled in the study. The final cohort of 170 patients was included for analysis.*


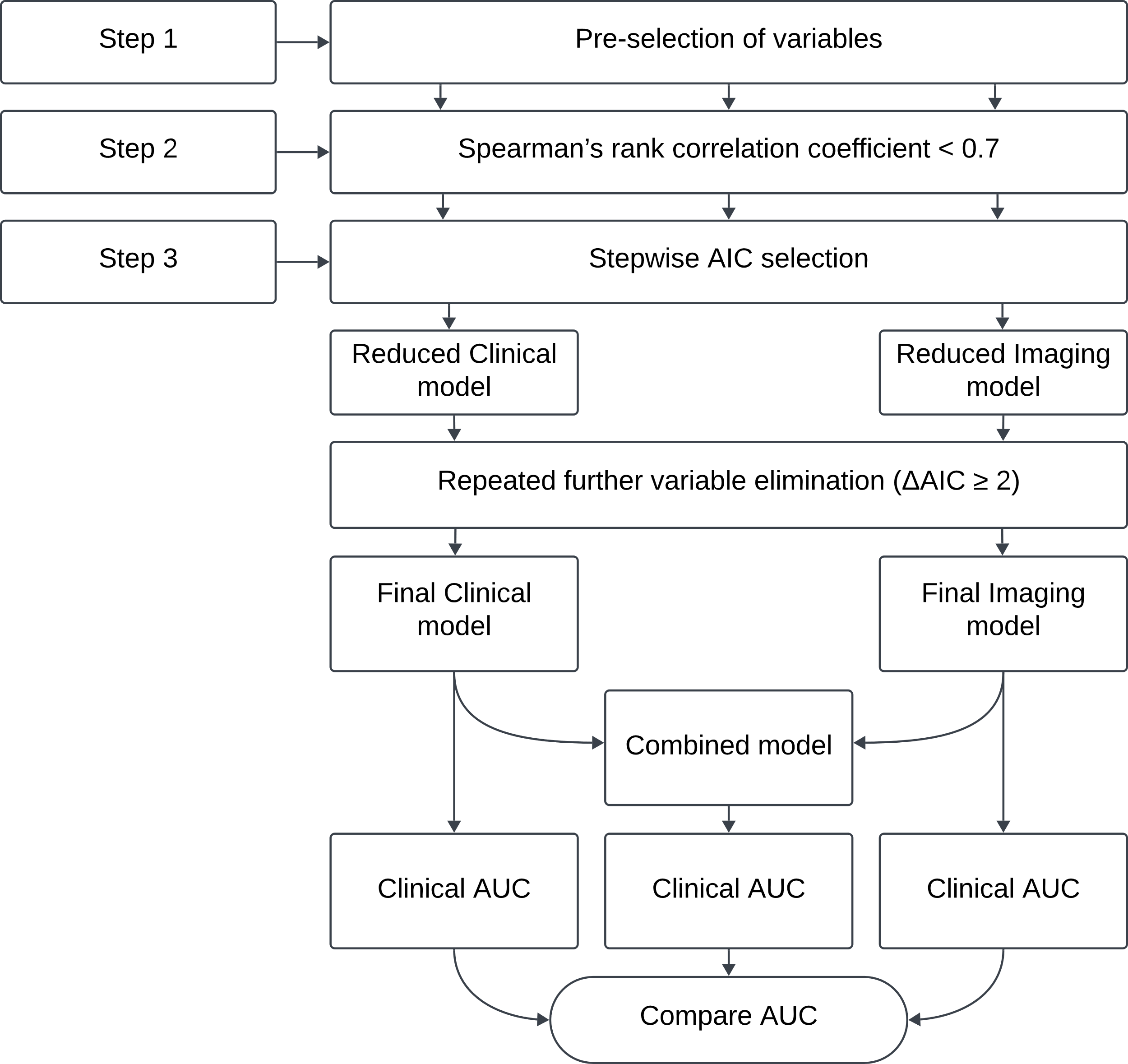


**Supplementary Figure 2: Methodology, variable selection and model building**

*Schematic visualization of the step-by-step variable selection process. AIC = Akaike information criterion.*

*
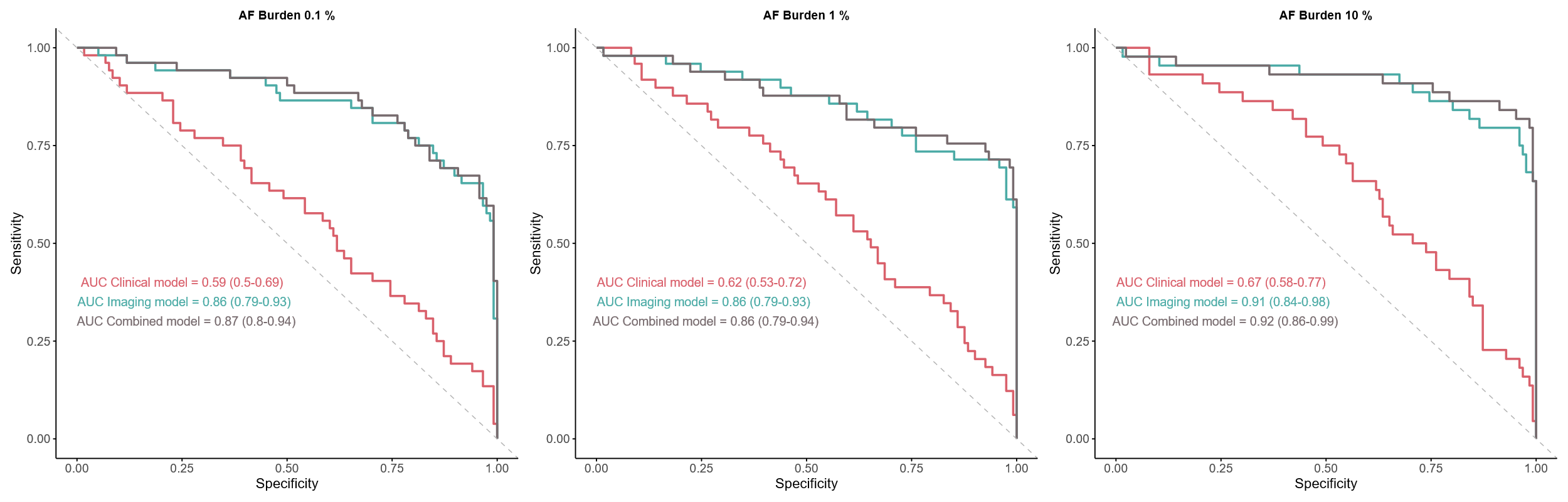
***Supplementary Figure 3: ROC curves of the clinical, imaging and combined model (for different cut-off’s 0.1%, 1% and 10%)**

*Receiver operating characteristic (ROC) curves for the clinical, imaging, and combined models at AF burden cut-offs of 0.1%, 1%, and 10% (left to right). The clinical and combined models were adjusted for age and sex. Model performance is expressed as the area under the curve (AUC) with 95% confidence intervals.*
